# The Rise of Contrast-enhanced Roentgenology: An Illustrated and Chronological Overview

**DOI:** 10.5334/jbr-btr.1222

**Published:** 2016-11-19

**Authors:** René Van Tiggelen

**Affiliations:** 1 Belgium Museum for Radiology, BE

**Keywords:** History, Vascular radiology, X-ray

## Abstract

Contrast opacification of the vascular system has become the mainstay of radiological examinations. This brief illustrated and chronological historical overview commences with the vascular opacification on a corpse via direct puncture of the aorta at both the thoracic and the abdominal levels and ends with the analogous exams on computed tomography (CT) and magnetic resonance imaging (MRI) via peripheral venous puncture.

Victory has many fathers, but defeat is always an orphan.

—John F Kennedy (April 21, 1961)

## Introduction

To know where you go to, it helps to know where you come from. Contrast opacification of the vascular system has become the mainstay of radiological examinations. This brief illustrated and chronological historical overview commences with the vascular opacification on a corpse via direct puncture of the aorta at both the thoracic and the abdominal levels and ends with the analogous exams on computed tomography (CT) and magnetic resonance imaging (MRI) via peripheral venous puncture. The major improvements applied to both the equipment and the techniques over more than a century are highlighted, with a special emphasis for the people who have pioneered and modified these approaches. While therapeutic and diagnostic approaches are increasingly distinctive radiological domains, both highly rely on a precise anatomical assessment of macroscopic blood vessels and the microvasculature. The road from the simple radiographer to the actual interventional practitioner has been long, with intricate paths that mainly include the contrast media, the vascular access, and the image capture.

## Contrast Media

**Figure 1:**
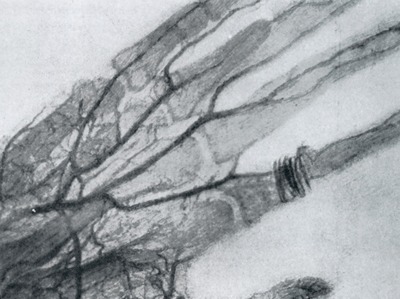
The world’s first angiogram of an amputated hand from a dead body [1].

In January 1896, Eduard Haschek and Otto Theodor Lindenthal of Vienna opacified—by means of a mixture of chalk, cinnabar (mercury sulfide), and Vaseline—the vessels of an amputated hand (Figure 1), and the concept of vascular contrast agent was born [1]. In many other countries, similar opacifications were performed on corpses by means of a mixture of Teichmann, composed of chalk, minium, and petroleum, or with X-ray opaque liquids of a closely comparable composition.

In France, in 1923, Jean Athanase Sicard (Necker Hospital, Paris) and Jacques Forestier (Aix-en-Provence) injected 5 cc of Lipiodol, an oil-based contrast agent, into the femoral vein to visualize the cardio-vascular system of a living dog. They observed that the contrast agent was scattered by the right ventricle and formed multiple pulmonary emboli that disappeared in 10–12 minutes [2].

**Figure 2:**
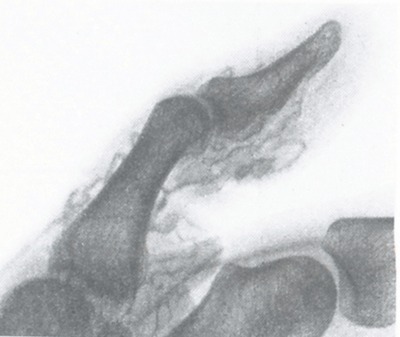
One of the first angiograms of a peripheral blood vessel of the thumb [3].

In Germany, in 1923, Joseph Berberich, of the Ear Nose Throat Department of Frankfurt am Main University, and Samson Hirsch, of the municipal hospital of the same city, performed the first angiography (Figure 2) on a living human with strontium bromide [3]. They injected, apparently without any problem, the contrast agent into an obstructed arm vein and observed stasis of the opaque material.

With time, only iodine, out of all the elements in the periodic table, was found suitable for injection into the circulation as a radiographic contrast agent. Although many elements are more radiopaque, none can currently be injected as safely as iodine into the circulation in sufficient concentrations and in sufficient dose to produce radio-opacity of diagnostic quality.

**Figure 3:**
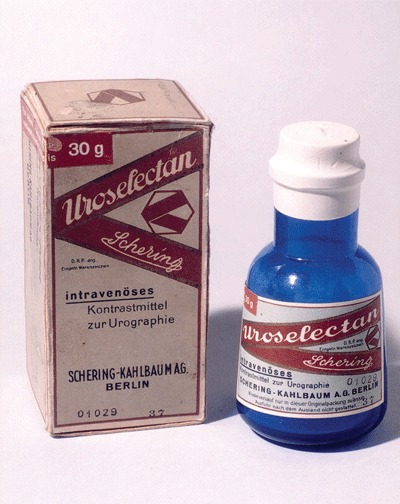
The best contrast medium at that time.

In the late 1920s, a 25 percent solution of thorium iodide (Thorotrast) was introduced as contrast agent. It was later withdrawn when its danger as a continuing source of internal radioactivity became apparent with the developments of tumors (thorotrastomas). In 1929, the first major achievement in the development of contrast media was the introduction by Moses Swick of iodides of the pyridine nucleus that were first used in excretory urography (Uroselectan, Schering) (Figure 3) [4].

The need for contrast agents suitable to more recent imaging techniques such as ultrasound (US) and magnetic resonance imaging (MRI) has led to the development of microbubbles and gadolinium/manganese-based contrast agents, respectively. The bio-distribution of MRI contrast agents parallels those of X-ray contrast agents, as they diffuse to the interstitial space from the vascular space and vice versa, whereas US microbubbles are limited to the vascular space. Current research efforts are directed towards increasing these contrast agents’ specificity for diseases.

## Vascular Access (From Puncture to Catheterism)

**Figure 4:**
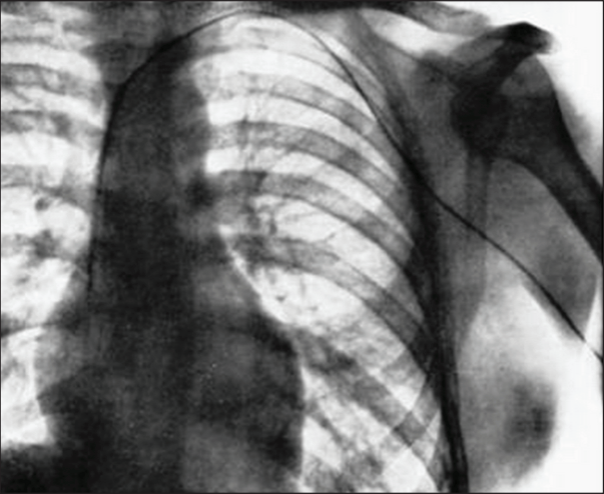
Radiography of the heart, catheterized by W. Forssman in Eberswalde (Germany) [5].

In 1929, Werner Forssmann, working at the Surgery Hospital of Berlin, performed the first cardiac catheterization on himself (Figure 4) [5]. He inserted a plastic tube in his cubital vein and guided it, with the cooperation of his nurse, to the right chambers of his heart. He showed the importance of this method by using uroselectan to visualise the pulmonary arteries later in 1931. As his scientific work was only moderately appreciated by his colleagues, he returned to surgery. André Cournand (Columbia University, New York) and Dickinson Richards (BelleVue Hospital, New York) revisited Forssmann’s technique to develop clinical cardiac catheterization. The Nobel Prize in Physiology or Medicine of 1956 rewarded the work of these three physicians.

In 1931, Egas Moniz, Lopo de Carvalho, and Almeida Lima, from Lisbon, published an article titled “Angiopneumography”, a term which they applied to techniques of opacification of the pulmonary vessels by the means of sodium iodine [6].

**Figure 5:**
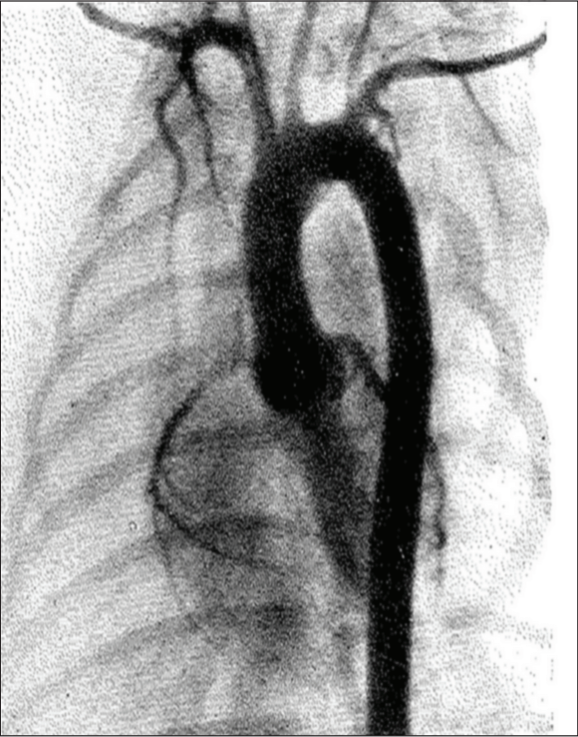
Retrograde catheterization of the aorta in a rabbit [7].

In 1933, Peter Rousthöi, at the University of Stockholm, established and published a new technique of angiocardiography via retrograde catheterization (Figure 5) of the aorta on a rabbit and an ape in which he passed the catheter through the aortic valve to reach the left ventricle [7].

**Figure 6:**
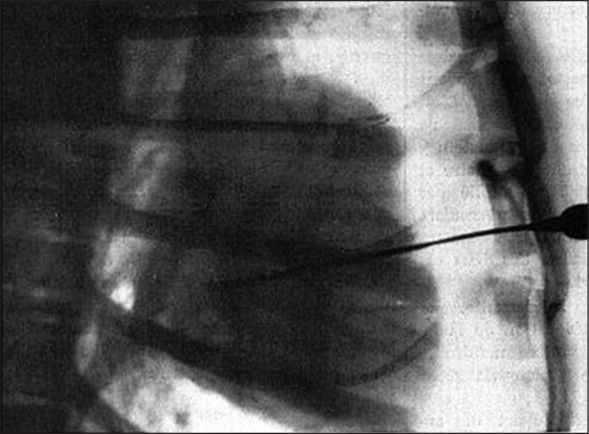
Needle in situ for the intraventricular injection on a dog [8].

That same year, Henri Reboul (trainee of Professor Faures-Frémie, Collège de France, Paris) and Maurice Racine (cardiologist at the Saint Maurice Hospital in Paris) studied, through trans-ventricular puncture (Figure 6) of an anaesthetized dog, the different phases of the cardiac cycle, the aorta, and, in some cases, the atrium and the left pulmonary veins after injection of Ténébryl, a then new iodine contrast [8].

In 1936, direct puncture of the thoracic aorta was first performed on humans by Innocenzo Nuvoli (surgeon-in-chief of the hospitals of Rome), who succeeded in demonstrating aneurysms, tortuosity, and other abnormalities. He utilized a bone marrow needle to perforate the skin, the subcutaneous tissue, and the sternum in the midline at the level of the second intercostal space [9].

**Figure 7:**
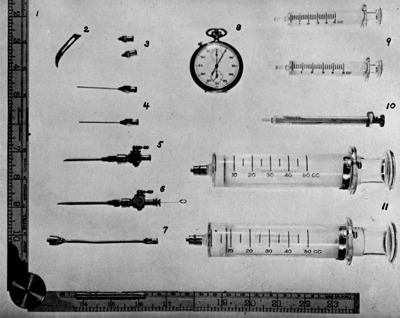
Essentials for arteriography. Note that the chronometer measures circulation time by intravenously injecting 5 cc of ether in 20 cc of saline and by detecting the odor in the patient’s breath [10].

In 1938, George Porter Robb, of the College of Medicine University of New York, and Israël Steinberg, of Harvard University, described a new method of angiocardiography. They used ether and cyanide circulation time as a guide (Figure 7) for timing successive exposures to obtain sequential opacification of the heart [10].

**Figure 8:**
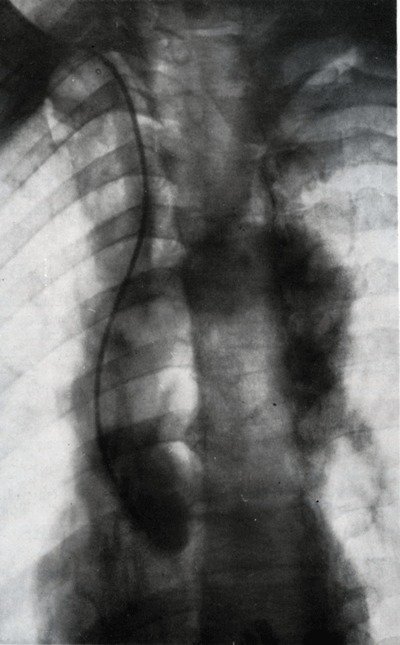
Catheter introduced into the aorta. The ascending aorta, the arch with all the brachiocephalic branches, and the descending aorta are visualised [12].

**Figure 9:**
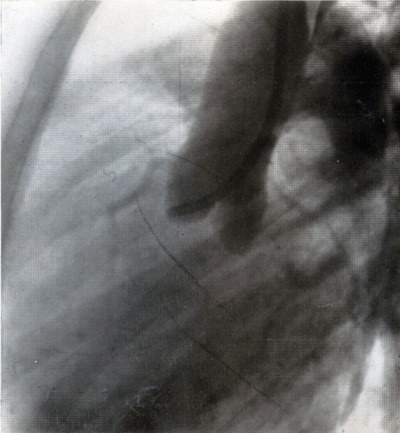
The sinus of Valsalva and semilunar valves are clearly seen. The two coronary arteries are also visible [13].

A decade later, in 1948, Stig Radner (University of Lund, Sweden) published a new technique (Figure 8): the thoracic aortography through the catheterization of the radial artery [11,12]. The same year, Gunnar Jönsson (Södersjukuset Hospital, Stockholm) described coronography in a human by injecting the contrast medium through a catheter induced into the aorta (Figure 9) [13].

In 1953, the revolutionary development that paved the way for the modern arteriography was the description of a new percutaneous catheter technique introduced by Sven Ivar Seldinger at Karolinska Hospital [14].

In 1958, Mason Sones Jr, a pediatric cardiologist at the Cleveland Clinic, accidentally discovered that he could inject directly into the coronary arteries and obtain good images. Thus, he made one of the greatest discoveries in the history of angiography and became the father of selective coronary arteriography. In the Sones technique, the catheter is guided towards a coronary artery orifice by the curvature of the aorta [15]. However, when the aorta is dilated, guidance remains uncertain.

**Figure 10:**
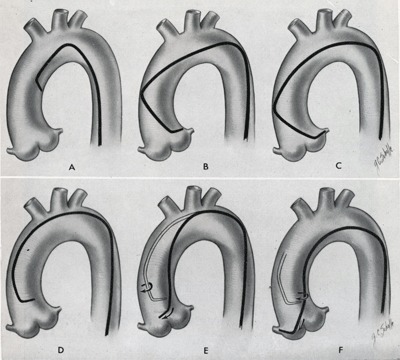
Diagram illustration of a left and a right coronary catheterization [16].

**Figure 11:**
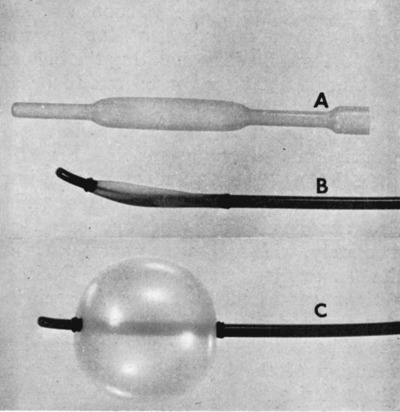
Distal end of a balloon catheter. Dipped latex balloon with expanded midsection for human use (A). Assembled catheter with constant cross-section balloon for axial use (B). Animal catheter with balloon fully inflated (C) [17].

**Figure 12:**
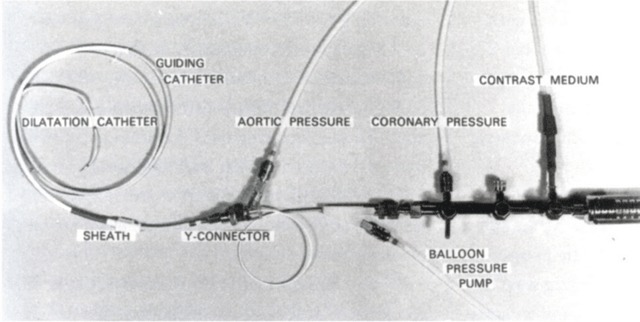
Grüntzig’s balloon-dilatation catheter and original equipment [19].

In 1967, Melvin Judkins (University of Oregon Medicine School, Portland) set out to develop a simpler and more reliable catheterization method (Figure 10) that could be applied to both the dilated and the normal aorta [16]. Building on the pioneering work of Charles Dotter from Portland, Oregon (Figure 11) [17], Andreas Grüntzig (University Hospital of Zurich), starting in 1977, extended coronary catheterization to therapeutic uses (Figure 12) [18,19].

Lastly, the important role played by the industry designing the catheters that later allowed for the development of interventional procedures such as embolization and drug infusion should be examined [20].

## Image Capture and Other Technical Improvements

**Figure 13:**
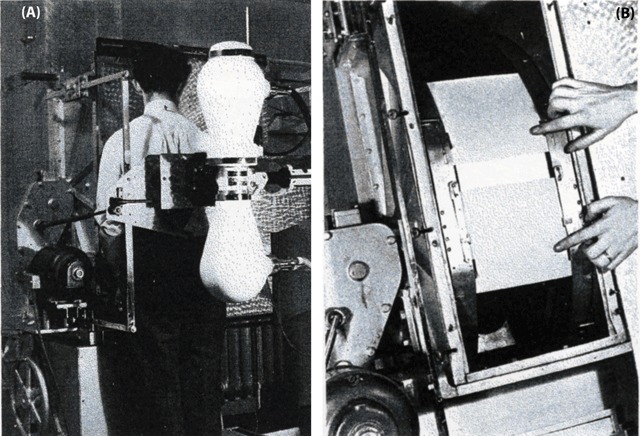
Direct radio-cinematography. By means of a powerful high-tension generator having a constant throughput, a fluorescent screen is illuminated, and with a motion picture apparatus, it was possible to produce on a small-size film (12 × 14 cm) at least 16 pictures per second. General view (A). Detail of the Gevaert’s applied film (B) [21].

The advent of radio-cinema is an important milestone in vascular imaging. A gastro-intestinal radiologist from Brussels, Dr. Van de Maele, developed an apparatus between 1935 and 1938 that allowed 16 radiographic images per second (Figure 13). This format however was still too limited for real-time angiography [21,22].

**Figure 14:**
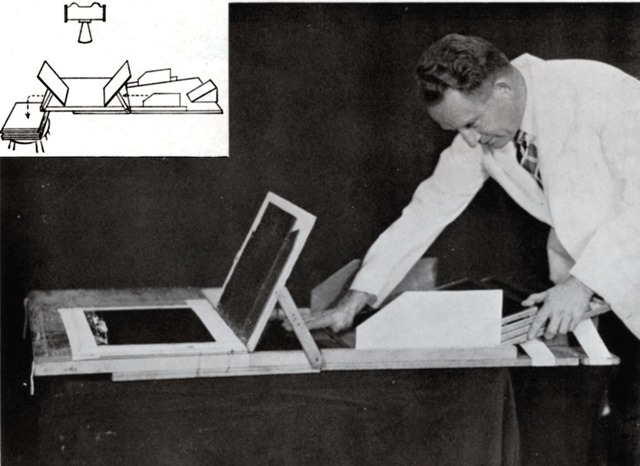
The diagram shows the approaching board with angle board, side guides, lead shield for film, and personal protection [23].

In 1949, in California, Merell Sisson (Stanford University Hospital) proposed a simple and manual system of a radiological cassettes changer for arteriography (Figure 14) that would be used by the American medical service until the Vietnam War because it never showed failure in operation [23].

**Figure 15:**
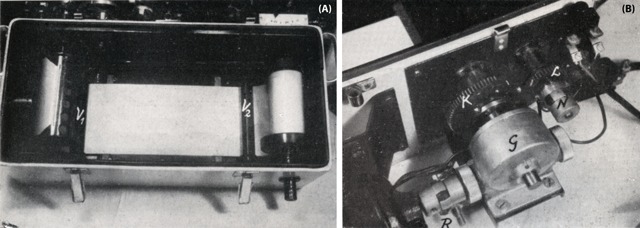
Film inserted in apparatus (A). Close-up driving mechanism (B) [24].

In Sweden, angiography was emphasised from the very beginning. In 1949, Ake Gidlund of the St Eriks Hospital (Stockholm) developed an apparatus (Figure 15) that allowed the use of large format roll film (12 × 12 first, then 28 × 28 inches) [24]. The first cassette changer had already appeared in 1946. It was later manufactured by the Georg Schönander Society according to the requirements established by Oliver Axén and John Lind. This technique, thanks to a novel fast changer (one-half second of interval) of cassettes, allowed simultaneous biplane arteriography [25].

**Figure 16:**
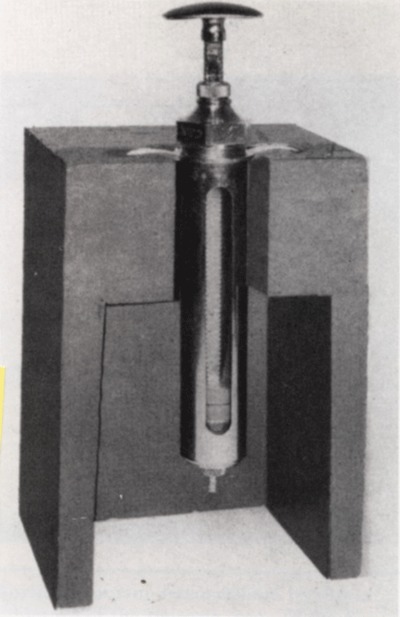
Manual injector of contrast media [26].

In 1957, Alan Thal and colleagues described a manually compressed injector (Figure 16) with a stainless-steel syringe that could be positioned in a vertical position, which facilitated the addition of a valve for removal at the top of the syringe [26].

**Figure 17:**
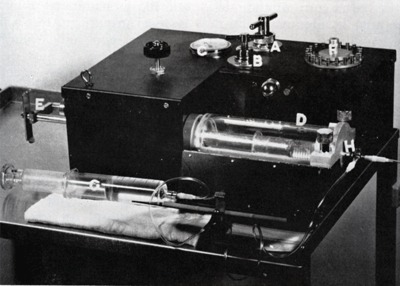
Vascular gas-powered injector for any type of angiographic work [27].

**Figure 18:**
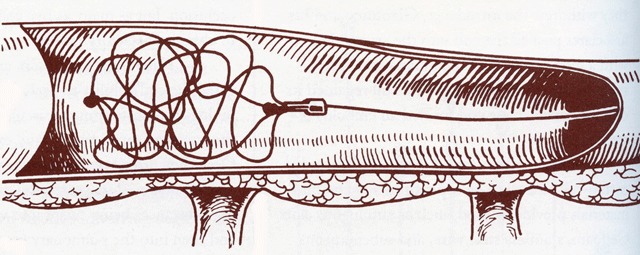
This prototype was a forerunner of the bird’s nest filter [28].

In 1960, Kurt Amplatz (University of Minnesota Medical School, Minneapolis) described a cardiovascular injector powered by carbon dioxide cartridges (Figure 17), such as those commonly used for the preparation of carbonated beverages. The major advantage of this system is that the injector weighted only 5 kg (11 lbs) [27]. For more years, Cesare Gianturco’s creative imagination (Texas Medical Center, Houston) gave rise to a wide variety of original interventional techniques and devices, including the cotton-tail embolus, wool coils, the bird’s nest filter (Figure 18), expandable stents, and the first balloon catheter [28].

## Today

For decades, only a catheter-based angiography was able to display accurate anatomic detail of the blood vessels for both diagnosis and therapy. Today, computerized tomography (CT), magnetic resonance imaging (MRI), and ultrasound (US) often serve as definitive angiographic studies, replacing invasive catheterization. In most cases, using these techniques only requires a peripheral venipuncture. With these techniques, interest has also shifted from macroscopic angiography to microvasculature, with the aim to precisely describe the distribution of the contrast agent at a microscopic level. This enables, for example, the evaluation of tumour microvasculature at different locations and in response to specific therapeutic agents to refine the treatment and the prognosis of patients with cancer, as these responses are far more sensitive than the change in size.

Recent improvements with all these techniques have gone towards better tissue characterization and faster scanning and processing times. For example, during the past decade, CT has seen the development of several technologies, such as the slip-ring gantry, the multi-row detector arrays, high-load X-ray tubes, and faster computers. Altogether, these progresses enabled dual-energy scanning, which allows extracting and reconstructing for the spectra related to each element of the periodic table. Applying this to iodine realistically paves the way not only for the easiest non-invasive angiographies, but also advanced applications such as perfusion. While we are about to reach this level of sophistication and comfort for the patient and the staff, it has to be stressed that our ancestors were pioneers who deserve our full consideration and respect.
